# Cyclisation Increases the Stability of the Sea Anemone Peptide APETx2 but Decreases Its Activity at Acid-Sensing Ion Channel 3

**DOI:** 10.3390/md10071511

**Published:** 2012-07-16

**Authors:** Jonas E. Jensen, Mehdi Mobli, Andreas Brust, Paul F. Alewood, Glenn F. King, Lachlan D. Rash

**Affiliations:** The University of Queensland, Institute for Molecular Bioscience, Brisbane, QLD 4072, Australia; Email: eklund_jensen@hotmail.com (J.E.J.); m.mobli@uq.edu.au (M.M.); a.brust@imb.uq.edu.au (A.B.); p.alewood@imb.uq.edu.au (P.F.A.)

**Keywords:** sea anemone, peptide, APETx2, ASIC3, cyclisation, truncation, stability

## Abstract

APETx2 is a peptide isolated from the sea anemone *Anthopleura elegantissima*. It is the most potent and selective inhibitor of acid-sensing ion channel 3 (ASIC3) and it is currently in preclinical studies as a novel analgesic for the treatment of chronic inflammatory pain. As a peptide it faces many challenges in the drug development process, including the potential lack of stability often associated with therapeutic peptides. In this study we determined the susceptibility of wild-type APETx2 to trypsin and pepsin and tested the applicability of backbone cyclisation as a strategy to improve its resistance to enzymatic degradation. Cyclisation with either a six-, seven- or eight-residue linker vastly improved the protease resistance of APETx2 but substantially decreased its potency against ASIC3. This suggests that either the *N*- or *C*-terminus of APETx2 is involved in its interaction with the channel, which we confirmed by making *N*- and *C*-terminal truncations. Truncation of either terminus, but especially the *N*-terminus, has detrimental effects on the ability of APETx2 to inhibit ASIC3. The current work indicates that cyclisation is unlikely to be a suitable strategy for stabilising APETx2, unless linkers can be engineered that do not interfere with binding to ASIC3.

## 1. Introduction

The last few decades have seen a vast change in the pharmaceutical industry’s attitude towards peptide drugs. Historically, peptide therapeutics have been largely avoided, in part due to the high cost of production and their short half-lives but mostly due to their lack of stability and low oral bioavailability compared to small molecules. However, peptides can offer significant advantages over small molecules when it comes to therapeutic applications. Their high potency, exquisite selectivity (often imparted by millions of years of evolutionary refinement) and low chemical toxicity can result in safer molecules with fewer side effects [1]. Recent decreases in production costs, changing attitudes towards the utility of biologics for a broad range of therapeutic applications, the development of improved methods for enhancing peptide-drug stability and bioavailability, and increasing acceptance of non-oral delivery has resulted in a strong surge in the development of peptides as drug leads in the last twenty years [2]. There are now ~60 peptide drugs on the market which had combined sales in 2010 of $13 billion [3]. Despite the growing acceptance of peptide drugs, the development of peptides with promising biological activity into good drug candidates faces many challenges, with *in vivo* stability being one of the major issues that must be overcome.

A drug that is delivered orally needs to pass through a series of often hostile environments on its way to the bloodstream and site of action. In the digestive system it encounters an acidic stomach then a neutral to alkaline small intestine, both of which contain a battery of proteolytic enzymes [4]. Once in the bloodstream, the drug must resist degradation in the liver (hepatic clearance or first-pass metabolism), be stable in plasma, and avoid being rapidly cleared by glomerular filtration in the kidneys. Rapid degradation and clearance can severely limit a peptide’s application as a therapeutic and hence improving its biological half-life without losing activity and specificity is highly desirable. There are numerous options for improving the stability and potential oral bioavailability of peptides. These include substitution of L-amino acids with D-amino acids, addition of polyethylene glycol chains (PEGylation), modification of the *N*- and/or *C*-termini, and reducing the flexibility of the peptide structure. An efficient way of achieving the last two strategies simultaneously is by cyclising the backbone of the peptide. Cyclisation of the peptide backbone has previously been reported to limit enzymatic degradation without affecting peptide potency and specificity [5,6,7]. 

Recent technological advances are finally allowing the enormous diversity of venom peptides to be exploited in the search for novel bioactive molecules [2]. There are currently six FDA-approved drugs derived from venom proteins, with a further ten in clinical trials and many more in various stages of preclinical development [2]. Venoms from snakes, scorpions, spiders, cone snails and sea anemones, amongst others, are being screened to identify potent and selective therapeutic leads, particularly targeting specific neuronal ion channels and receptors [8,9,10,11]. A recent outcome of such screening efforts is APETx2, a peptide isolated from the sea anemone *Anthopleura elegantissima* which is the only known selective blocker of acid-sensing ion channel 3 (ASIC3) [12].

ASICs are non-voltage gated, pH-sensitive sodium ion channels. They are distributed in the central and peripheral nervous system of chordates and play a role in pain perception amongst other biological functions [13]. ASIC3 in particular is highly expressed in peripheral sensory neurons and appears to be a key mediator of inflammatory pain. ASIC3 may also play a role in anxiety and cardiovascular function and insulin resistance [14]. APETx2 abolishes acid-induced pain in rats [15,16] and the peptide is in preclinical studies as a potential analgesic [17]. However, very little is known about the biological stability of APETx2 or the molecular basis of its interaction with ASIC3.

Cyclisation of the peptide backbone is an efficient method of increasing the protease resistance of peptides and it can often be achieved without loss of activity. Disulfide-rich peptide toxins are especially suitable for cyclisation as the cysteine residues can be exploited in the cyclisation process using native chemical ligation (NCL) [18]. APETx2 is a tightly folded peptide of 42 residues, six of which are cysteines. Furthermore, the solution structure of APETx2 [19] indicates that the *N*- and *C*-termini are relatively close in space, making APETx2 a good candidate for cyclisation to improve stability. We previously reported an efficient synthetic strategy for production of APETx2 [18]. In this study we describe the backbone cyclisation of APETx2 and the effect it has on its biological stability and functional activity at ASIC3. We also investigated the functional role of the *N*- and *C*-termini of APETx2 by making and testing the activity of *N*- and *C*-terminally truncated versions of the peptide. To our knowledge, this is the first time a sea anemone venom peptide has been cyclised, and the first report of the functional importance of the peptide termini for this novel family of marine-derived peptides.

## 2. Results and Discussion

### 2.1. Peptide Design and Synthesis

Investigation of the ensemble of solution structures of APETx2 (Protein Data Bank ID: 1WXN) revealed a distance between the *N*- and *C*-termini of 10.8 ± 2.4 Å. Based on the findings by Clark and colleagues [20], it was estimated that six residues or more would be needed to span the gap between the two termini. Thus, three cyclic analogues were designed in which the termini were joined with a linker comprising six (GASGSA, cAPETx2_6RL), seven (AGASGSA, cAPETx2_7RL) or eight residues (SAGASGSA, cAPETx2_8RL). Gly, Ala and Ser residues were used in the linker as their side chains are small, and the linker sequence was designed to be non-repetitive in order to facilitate NMR analysis to examine the structural integrity of the cyclic peptides. To further elucidate the contribution of the *N*- and *C*-termini to the activity of APETx2, truncated peptides were designed: two residues were deleted from the *N*-terminus (APETx2_3–42) and three residues were removed from the *C*-terminus (APETx2_1–39). 

**Figure 1:**
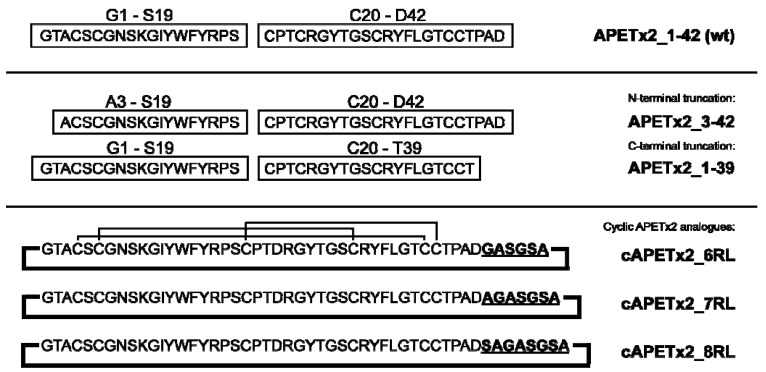
Wild-type APETx2 and truncation analogues were made in a two-step approach whereby the two peptide chains were synthesised separately then joined via NCL. The cyclic analogues were synthesised as single peptide and cyclised using NCL.

Non-cyclic versions of APETx2 were made using two short fragments that were joined by NCL to produce the full-length peptide, which was subsequently oxidised to form the three intramolecular disulfide bonds. The cyclic analogues were synthesised as single, full-length peptides. In this case, NCL was used to cyclise the peptide backbone of the reduced, linear peptides. Cyclisation and oxidation was done as a one-step reaction using the same oxidation buffer and approach as for the non-cyclic APETx2 variants. The sequences of wild-type APETx2 (APETx2_wt) and the analogues made are shown in Figure 1, along with a summary of the synthetic strategy used to produce them. Despite using a folding condition previously shown to result in the native disulfide isoform of APETx2 (there are 15 isoforms possible) [18], oxidation of the cyclic versions resulted in 4–5 isoforms; the dominant isoform was the native fold, but it required several rounds of purification. 

### 2.2. Confirmation of Structural Integrity by NMR

2D ^1^H-^15^N NMR experiments (Selective Optimised Flip-Angle Short-Transient heteronuclear multiple quantum coherence; SOFAST-HMQC) were acquired to assess the structural integrity of APETx2 analogues. The chemical shift of ^1^H_N_ and ^15^N nuclei are exquisitely sensitive to their local electronic environment [21] and therefore both local effects and major structural changes can easily be assessed by comparing the HMQC spectrum of analogues with that of the native peptide [22]. As APETx2 and derivatives were synthesised without the use of isotope-labelled amino acids, the heteronuclear correlation experiments relied on the natural abundance of ^15^N (0.4%), making them inherently insensitive. In order to improve sensitivity, we used the SOFAST-HMQC approach [23] in combination with an ultra-high field NMR spectrometer (900 MHz), which allowed us to acquire high quality natural abundance ^1^H-^15^N spectra in ~2–12 h depending on peptide concentration. 

**Figure 2:**
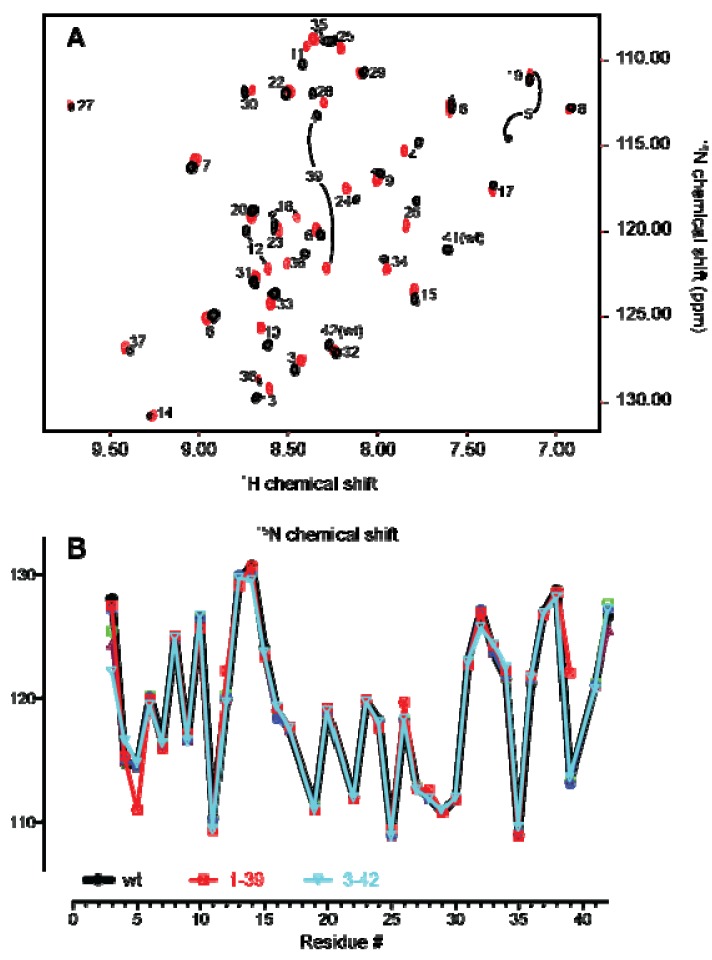
(**A**) SOFAST-HMQC spectrum of APETx2_wt (black) overlaid with that of APETx2_1–39 (red). A crosspeak is present for each H-N bond (*i.e.*, for each non-proline residue), including non-labile sidechain NH groups (Trp Nε-Hε, Asn Nδ-Hδ2, Gln Nε-Hε and Arg Nε-Hε). (**B**) Backbone ^15^N chemical shifts for APETx2_wt (black), APETx2_1–39 (red) and APETx2_3–42 (cyan).

The HMQC spectrum of APETx2_wt was assigned based on nuclear Overhauser effect spectroscopy (NOESY), total correlation spectroscopy (TOCSY) and double quantum filtered correlation spectroscopy (DQF COSY) spectra (data not shown). Figure 2A shows an overlay of the SOFAST-HMQC spectra of APETx2_wt and APETx2_1–39, which highlights any structural perturbations caused by the *C*-terminal truncation. The most profound chemical shift changes were observed for Thr39, which was expected as its chemical environment changes dramatically due to the removal of Pro40, Ala41 and Asp42, making it the *C*-terminal residue. The next most noticeable shifts occur for Ser5 and Ile12. Ile12 is in close proximity to Ala41 and Asp42 positioned between the *N*- and *C*-termini, which explains this perturbation. The side chain of Thr39 is in direct contact with the peptide backbone at Ser5; thus, the marked change in the chemical environment of Thr39 likely results in the observed chemical shifts for Ser5. However, in general, the majority of crosspeaks in the HMQC spectra of both truncations overlay well with the wild-type peptide, as indicated by the overlay of ^15^N chemical shift values for wild-type APETx2 and the two truncated peptides (Figure 2B). We therefore conclude that removal of residues 1–2 or 40–42 causes minimal perturbation of the APETx2 structure.

**Figure 3:**
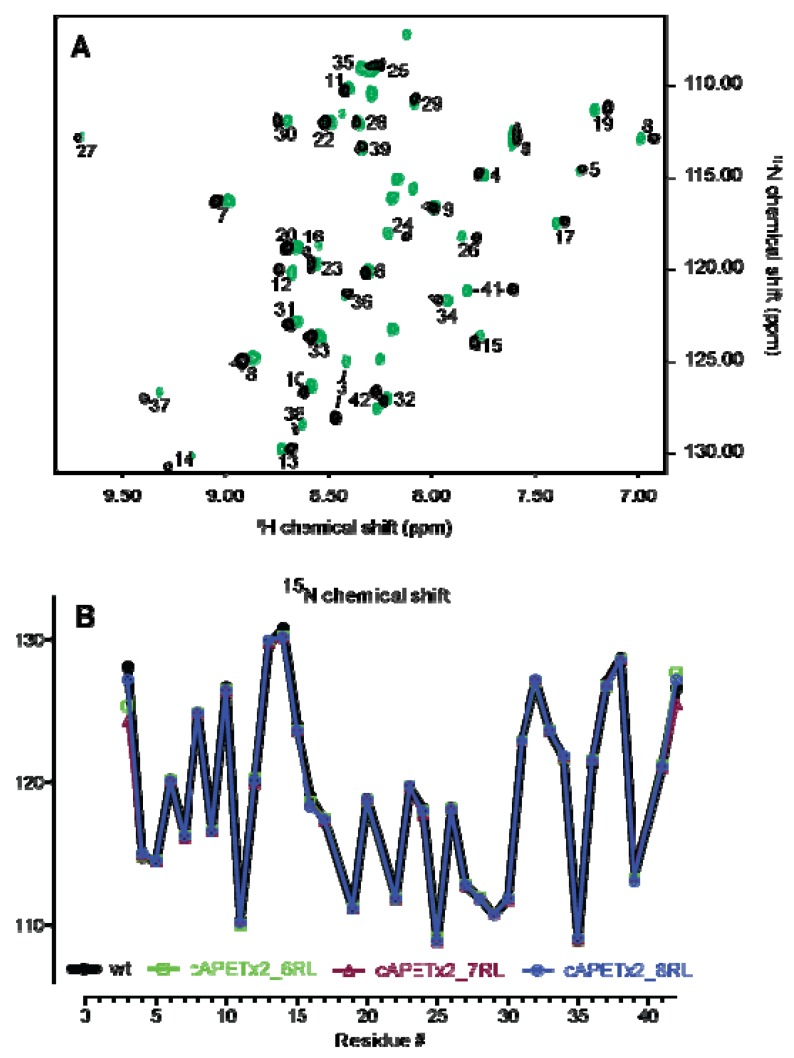
(**A**) Overlaid HMQC spectra of APETx2_wt (black) and cAPETx2_6RL (green). (**B**) Overlay of the backbone ^15^N chemical shifts of APETx2_wt (black) and cyclic APETx2 with either a 6-residue (green), 7-residue (maroon), or 8‑residue (blue) linker.

The structural integrity of the cyclic analogues was assessed in the same way. Figure 3A shows an overlay of the SOFAST-HMQC spectra of cAPETx2_6RL and APETx2_wt. Eight residues in the HMQC spectrum of cAPETx2_6RL are not assigned and correspond to Gly1 and Thr2 and the linker GASGSA. The chemical shifts of the linker residues all appear in the disordered region, indicating that it has no particular secondary structure. Crosspeaks for Gly1 and Thr2 are visible in the cyclic analogues and not the truncation analogues, as in the non-cyclic peptides the H_N_ protons of these residues are in fast chemical exchange with the solvent. As expected, addition of a linker caused minor chemical shift changes for the terminal residues Ala3, Ala41 and Asp42. As shown in Figure 3B, the backbone ^15^N chemical shifts for the cyclic peptides overlay extremely well with those of the wild-type toxin, indicating that cyclisation does not perturb the overall structure of APETx2. Therefore, any change in activity observed for the cyclic analogues is likely to be a direct consequence of the added linker residues rather than the result of a major structural perturbation. 

### 2.3. Biological Stability of Cyclic APETx2 Analogues

To assess the effect of cyclisation on the protease resistance of APETx2 the native toxin and cyclic analogues were compared in two stability assays: a tryptic digestion assay and a simulated gastric fluid (SGF) assay (*i.e.*, pepsin at low pH). Trypsin is a relatively specific protease that can potentially cleave APETx2 on the *C*-terminal side of Lys10, Arg24 and/or Arg31. Pepsin, on the other hand, is much less specific, cleaving between hydrophobic and preferably aromatic residues; it can potentially cleave APETx2 at 11 positions (*i.e.*, the carboxyl side of Ala3, Ile12, Tyr13, Trp14, Phe15, Tyr16, Tyr26, Tyr32, Phe33, Leu34 and Ala41). Thus, pepsin provides a more challenging test of peptide stability. Addition of the linker sequence did not add any more potential cleavage sites for either trypsin or pepsin. 

**Figure 4:**
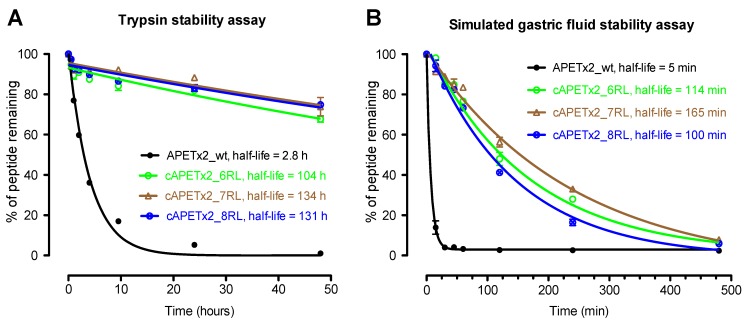
Decay curves showing the stability of APETx2_wt (black), cAPETx2_6RL (green), cAPETx2_7RL (brown), and cAPETx2_8RL (blue) against proteolytic cleavage by (**A**) trypsin and (**B**) pepsin.

Despite being a tightly folded, disulfide-stabilised peptide, wild-type APETx2 was not particularly resistant to either trypsin or SGF, with a half-life of ~2.8 h (Figure 4A) and ~5 min (Figure 4B), respectively. However, all three cyclic analogues were much more stable than the non-cyclic native toxin in both stability assays. Only ~4% of wild-type APETx2 remained after 48 h in the presence of trypsin, whereas ~70%–80% of the cyclic versions were still intact (Figure 4A). Similarly, wild-type APETx2 was completed digested after 2 h in SGF, whereas ~50% of the cyclic analogues remained intact (Figure 4B). These two stability assays clearly show that backbone cyclisation substantially improves the protease resistance of APETx2.

### 2.4. Activity of Cyclic APETx2_6-, 7- and 8-Residue Linker

**Figure 5:**
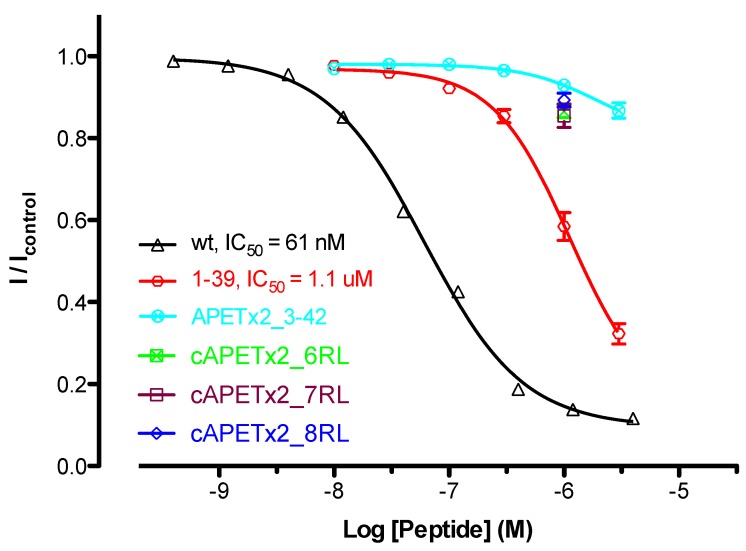
Comparison of the activity of wild-type APETx2 with cyclic and truncation analogues. Concentration-effect curves are shown for inhibition of rASIC3 currents by APETx2 wt (black), APETx2_1–39 (red) and APETx2_3–42 (cyan) (*n* = 6). The activity of the cyclic APETx2 analogues is shown only at 1 µM (*n* = 6).

The biological activity of wild-type APETx2 was compared with that of truncations and cyclic analogues using voltage-clamp electrophysiology experiments on *Xenopus laevis* oocytes expressing homomeric rat ASIC3. Compared to native APETx2, the activity of the three cyclic analogues was greatly reduced. At 1000 nM, a concentration of APETx2 that normally causes >90% inhibition of ASIC3 [18], all three cyclic analogues had little activity (~10% inhibition, *n* = 6) (Figure 5). Although a full concentration-effect curve was not obtained, there is an approximate 125-fold decrease in activity for all three cyclic analogues based on the concentration that causes 10% inhibition (~8 nM for wild-type APETx2 *vs*. 1000 nM for cyclic APETx2).

### 2.5. Activity of APETx2 *N*- and *C*-Terminal Truncations

With the exception of Arg17, which when mutated to alanine resulted in a 25-fold decrease in activity of APETx2 at mouse ASIC3 [24], nothing is known about the residues that contribute to APETx2’s inhibition of ASIC3. In particular, nothing is known about the role played by the *N*- and *C*-terminal residues in APETx2 activity. The substantially decreased activity of the cyclic APETx2 analogues indicates that the addition of a linker sterically impedes APETx2 binding or it abrogates important interactions between the channel and *N*- and/or *C*-terminal residues in APETx2. In order to test the latter hypothesis, we decided to determine the importance of the *N*- and *C*-terminal residues by making truncation mutants. Removal of the three *C*-terminal residues increased the IC_50_ value for APETx2 inhibition of ASIC by 18-fold from 61 nM to 1.1 μM (Figure 5). Due to the markedly lower activity of the *N*-terminal truncation analogue, it was not possible to obtain a full concentration effect curve (as there was insufficient material to test at the required high concentrations). However, at a concentration of 3 μM, the *N*-terminal truncation inhibited ASIC3 currents by only ~13% compared to the concentration of wild-type APETx2 required to cause a similar level of inhibition (~10 nM). This suggests that removal of the two *N*-terminal residues results in a 300-fold decrease in activity (*i.e*., a 16-fold greater loss of activity than caused by the *C*-terminal truncation). Thus, we conclude that interfering with either the *N*- or *C*-terminus of APETx2 results in a substantial loss in activity, although the *N*-terminal residues appear to be significantly more important.

### 2.6. Discussion

The sea anemone venom-peptide APETx2 is the most potent and selective inhibitor of ASIC3 discovered to date, making it a promising lead compound for the treatment of chronic inflammatory pain [13,25]. APETx2 provides effective analgesia when administered peripherally in multiple rodent pain models [15,16,26], making it uniquely interesting from both a research and drug development perspective. A recent study revealed that APETx2 also inhibits voltage-gated sodium channel (Na_V_) 1.8, another novel pain target, which may contribute to its analgesic activity [27]. Indeed, APETx2 in its native form is already in preclinical development as an analgesic drug. Thus, it is important to understand the factors affecting the biological stability of APETx2 and how they might be manipulated in order to improve its *in vivo* stability.

APETx2 is structurally related to the sea anemone toxins APETx1, BDS-I, BDS-II, BcIV and Am II, which are all grouped in the defensin family [19,28]. These toxins share the same disulfide connectivity (I–V, II–IV and III–VI) as well as a similar 3D structure characterised by a compact disulfide-rich core and a three- or four-stranded antiparallell β-sheet. Despite similarities in primary and tertiary structure, the abovementioned sea anemone toxins target different ion channels. APETx1, BDS-I and BDS-II block voltage-gated potassium channels, whereas BcIV blocks Na_V_ channels. Am II is paralytic to crabs, but its molecular target has not yet been identified [29]. Despite the variety of interesting ion channels targeted by this family of sea anemone peptides, nothing is known about their structure-activity relationships or biological stability. 

Despite having good solution stability under lab storage conditions (APETx2 is stable for months when dissolved in water and kept at 4 °C; unpublished observations) we found that wild-type APETx2 is highly susceptible to degradation by trypsin and pepsin, two proteolytic enzymes encountered in the digestive system. We therefore examined whether backbone cyclisation could be used to improve the biological stability of APETx2. Naturally occurring cyclic peptides such as plant cyclotides and sunflower trypsin inhibitor-1 are known to be highly protease resistant and these and other cyclic scaffolds are being exploited by peptide scientists to engineer improved stability into small peptides of pharmacological and therapeutic interest [30,31]. For example, backbone cyclisation substantially decreased the protease susceptibility of the small conotoxins MrIA and Rg1A without compromising their biological activity [32,33]. Moreover, cyclic Vc1.1 was shown to be orally active as an analgesic, whereas the non-cyclic, wild-type peptide was not [20].

APETx2 is highly amenable to backbone cyclisation as the *N*- and *C*-termini are spatially proximal, being separated by only ~11 Å. Based on previous studies with conotoxins [20] we calculated that a linker peptide comprising six or more residues would be required to connect the *N*- and *C*-termini. NMR structural studies showed that all three cyclic analogues had the same 3D fold as wild-type APETx2. Cyclisation vastly improved the protease resistance of APETx2, even in the extreme conditions of the simulated gastric fluid assay. The average time for passage of food through the stomach is 1–2 h [4]. Our results show that the native peptide is almost totally digested within 20 min in simulated gastric fluid, indicating that it would have very poor oral availability. However, after 2 h under the same conditions, the cyclic analogues were 40%–60% intact (an increase of 20–30 fold in their half life). Thus, in principal, backbone cyclisation could improve the stability of APETx2 enough to allow significant amounts of peptide to reach the small intestine where the majority of absorption occurs [4]. The improvement in stability in the presence of trypsin was even more remarkable (*i.e*., a ~40-fold increase in half-life from ~2.5 h to several days).

In spite of this promising increase in stability, the activity of APETx2 at ASIC3 was dramatically reduced by cyclisation. This was surprising for two reasons. First, the ASIC3-binding surface predicted from analysis of the structure of APETx2 [19], which is consistent with the only residue shown experimentally to be important for the interaction, namely Arg17 [24], suggests that connecting the *N*- and *C*-termini with a peptidic linker should not disrupt the interaction between APETx2 and ASIC3. Second, the study in which Arg17 was determined to be important for the interaction of APETx2 with ASIC3 used recombinant peptide (rAPETx2) that was expressed in yeast with an *N*-terminal His_6_ tag plus residual glutamate and phenylalanine residues from incomplete cleavage of the signal sequence; thus, the recombinant peptide had an eight-residue *N*-terminal extension (EFHHHHHH) [24]. This *N*-terminally-extended version of rAPETx2 retained its activity at mouse ASIC3 (IC_50_ of 37 nM), suggesting that the *N*-terminus either does not play a role in the ASIC3 interaction, or that addition of the eight residues does not perturb the interaction between native *N*‑terminal residues and the channel. However, our demonstration that cyclisation dramatically perturbs the activity of APETx2 suggests that either the *N*- or *C*-terminus, or both, *are* involved in its interaction with ASIC3.

We confirmed this hypothesis by showing that *N*- and *C*-terminal truncation analogues also had significantly diminished activity at ASIC3, with the *N*-terminus being more important (300-fold decrease in activity versus 18-fold decrease for the *C*-terminal truncation). At least some of the deleted residues (*i.e*., Gly1 and Thr2 at the *N*-terminus or Pro40, Ala41 and Asp42 at the *C*-terminus) must play an important role in the interaction of APETx2 with ASIC3, or alternatively the addition of a linker connecting the termini sterically impedes the toxin’s interaction with the channel. Given the much greater effect of removing the *N*-terminus than the *C*-terminus, it is likely that the *N*-terminus plays a direct role in the interaction of APETx2 with ASIC3 and that the reduced activity of *C*-terminally truncated APETx2 results from a relayed perturbation of the *N*-terminus. The two termini are spatially proximal (they are both in contact with Ile12), and thus it is possible that the removal of the three *C*-terminal residues has a detrimental effect on the orientation of the *N*-terminus, thereby causing a slight decrease in activity. If this is the case, adding a linker to connect the *N*- and *C*-termini may have resulted in sufficient perturbation of the *N*-terminus to disturb its interaction with ASIC3. Hence it may not be possible to engineer cyclic versions of APETx2 that retain activity unless a linker can be designed that has no effect on the terminal residues and/or has complementary interactions with ASIC3. Since the structure of ASIC3 has not been determined and the APETx2 pharmacophore is not known, such rational linker design is currently not feasible. 

**Figure 6:**
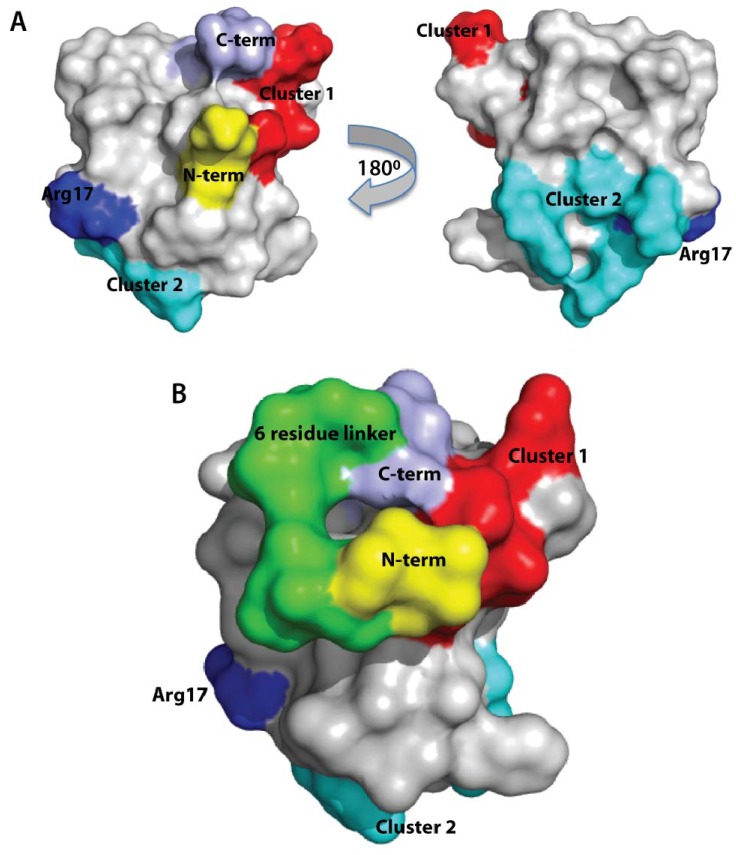
(**A**) Surface representation of APETx2 highlighting residues potentially involved in its interaction with ASIC3: Cluster 1 (residues A3, S5, N8, K10, T39 and A41) is coloured red and Cluster 2 (residues Y16, R17, P18, R31 and T36) is coloured cyan. The *N*- and *C*-termini are coloured yellow and violet, respectively. Arg17 is coloured dark blue. (**B**) Surface representation of homology model of cAPETx2_6RL showing the six-residue linker in green. Potential residues interacting with ASIC3 are highlighted as for (A).

Based on the solution structure of APETx2, Chagot and colleagues suggested two clusters of residues that could be involved in its interaction with ASIC3: Cluster 1 (A3, S5, N8, K10, T39 and A41) and Cluster 2 (Y16, R17, P18, R31 and T36) [19] (Figure 6A). The basis of their suggestion is that these residues differ between APETx2 and its paralog APETx1, an inhibitor of the hERG channel. As Cluster 1 and Cluster 2 are located on opposite faces of the toxin (Figure 6), it seems unlikely that ASIC3-interacting residues are found in both clusters since this would require the toxin to bind deeply in a pocket on the channel (an atypical mode of binding for peptidic ion channel modulators). Furthermore, this hypothesis ignores the possibility that the pharmacophore residues mediating the interaction with ASIC and hERG could partially overlap. The *N*- and *C*-termini are in close proximity to Cluster 1, however Arg17, which is part of the APETx2 pharmacophore [24], is close to Cluster 2. Our findings revealed that the *N*-terminus (which closer to Cluster 2 than the *C*-terminus) is likely to be directly involved in the interaction with the ion channel. Hence we suggest that residues in the *N*-terminus and most likely some of the residues in Cluster 2 (Y16, R17, P18, R31 and/or T36) form the pharmacophore on APETx2 that mediates its interaction with ASIC3.

Figure 6B depicts a homology model of cyclic APETx2 constructed using the structure of native APETx2 as a template (PDB coordinate file 1WXN); the six-residue linker is shown in green. Based on visual inspection of this model, it is clear that addition of a linker might disrupt important contacts between the *N*-terminus of APETx2 and ASIC3.

## 3. Experimental Section

### 3.1. Materials

N^α^-Boc-L-amino acids were from Merck Pty Ltd. (Kilsyth, Vic., Australia) except for N^α^-Boc-L-Trp(Hoc) that was purchased from Peptides Institute (Osaka, Japan). Resins and *S*-trityl-β-mercapto-propionic acid were purchased from Peptides International (Louisville, Kentucky, USA). *p*-Cresol, *p*‑thiocresol, 2‑(1*H*‑benzotriazol‑1yl)‑1,1,3,3‑tetramethyluronium hexafluorophosphate (HBTU), tris(2-carboxyethyl)-phosphine (TCEP), and 4-mercaptophenylacetic acid (MPAA) were purchased from Sigma Aldrich (Sydney, NSW, Australia). Diisopropylethylamine (DIEA) and trifluoroacetic acid (TFA) were from Auspep Pty Ltd. (Melbourne, Vic., Australia) and anhydrous hydrogen fluoride (HF) was purchased from BOC Gases (Sydney, NSW, Australia). Morpholinopropane sulphonic acid (MOPS) and tris(hydroxymethyl)amino-methane (TRIS) were from Astral Scientific Pty Ltd. (Sydney, NSW, Australia). Screw-cap glass peptide synthesis reaction vessels with sintered glass filter frit were purchased from Embell Scientific Glassware (Queensland, Australia). Trypsin, pepsin and pepstatin A were purchased from Sigma Aldrich (Sydney, NSW, Australia). D_2_O was from Novachem (Vic., Australia) and susceptibility-matched NMR cells with 5 mm outer diameter were purchased from Shigemi Inc. (Pennsylvania, USA). Centrifuge filters with a pore size of 0.22 μm were purchased from Millipore (Victoria, Australia). The mMessage mMachine cRNA transcription kit for making mRNA was purchased from Ambion Inc., (Austin, TX, USA), and capillary glass for oocyte injections and electrodes was purchased from SDR Clinical Technology (Sydney, NSW, Australia).

### 3.2. Peptide Production

#### 3.2.1. Peptide Synthesis

APETx2 truncation analogues were assembled using a stepwise *in situ* neutralisation protocol for *tert*-butoxycarbonyl (Boc) chemistry [34]. The peptides were synthesised on Boc-Gly-Pam polystyrene resin as previously described [18]. APETx2(Ala3–Ser19)-α-thioester and APETx2(Gly1–Ser19)-α-thioester were synthesised on Boc-Gly-Pam resin, while APETx2(Cys20–Asp42) and APETx2(Cys20–Thr39) were synthesised on Boc-Asp-Pam resin. 

Cyclic peptides were synthesised using the same stepwise *in situ* neutralisation protocol as described above for the truncation analogues: Peptides were attached to the PAM-Gly resin via a β‑mercaptopropionic acid linker and the 46, 47 and 48 amino acids added. Upon cleavage of the peptide chain from the resin, the linker generates a *C*-terminal thioester that facilitates the cyclisation during the NCL/oxidation reaction. 

Amino acid side-chain protection was as follows: Asn (Xan), Arg (Tos), Asp (OcHex), Cys (4-MeBzl), Lys (ClZ), Ser (Bzl), Thr (Bzl) Trp (HOC) and Tyr (BrZ). All syntheses were carried out on a 0.5 mmol scale using HBTU as the activation reagent, and DIEA as the neutralising base. Neat TFA was used to remove the Boc protecting group. Side-chain deprotection and cleavage from the resin were carried out in HF: *p*-cresol: *p*-thiocresol (9:0.5:0.5) for 1.5 h at −5 °C. HF was removed by evaporation under reduced pressure and peptides were precipitated and washed twice with cold diethyl ether, before being dissolved in 50% acetonitrile/water/0.1% TFA and lyophilised. 

#### 3.2.2. RP-HPLC and Mass Spectrometry

Crude cleaved peptides were purified by reversed-phase high performance liquid chromatography (RP-HPLC) using a Waters Delta Prep 3000 system connected to a Vydac C_8_ column (50 × 250 mm). The flow rate was 50 mL/min and the gradient used was 15%–25% solvent B (0.043% TFA in 90% acetonitrile) over 5 min, followed by 25%–45% solvent B over 40 min. Solvent A was 0.05% TFA in H_2_O. Preparative and analytical HPLC was performed on a Shimadzu Prominence HPLC system using Vydac C_18_ columns (TP218 series) of the following sizes and flow rates (22 × 250 mm, 8–10 mL/min; 10 × 250 mm, 4 mL/min; 4.6 × 250 mm, 1 mL/min; 2.1 × 250 mm, 0.25 mL/min). Peptide elution was monitored via absorbance at 214 and 280 nm. Peptide masses from HPLC fractions were determined using an API 2000 triple quadrupole mass spectrometer (Applied Biosystems, CA, USA).

#### 3.2.3. Cyclisation and Oxidation of APETx2 Analogues

Cyclisation and oxidation were carried out in a single step as follows. Reduced, linear peptide was dissolved in 6 M guanidine hydrocholride (GnHCl) at 10 mg/mL before being added to the freshly prepared oxidation buffer consisting of 0.126 M ammonium acetate, 2.5 mM reduced glutathione (GSH), 0.25 mM oxidized glutathione (GSSG), 0.5 M GnHCl and 50% methanol, pH 7.9 to a final peptide concentration of 0.1 mg/mL. The cyclisation and oxidation process was monitored using analytical RP-HPLC and ESI-MS. After 15 h the reaction was quenched by diluting the solution to less than 25% methanol with solvent A, then the mixture was syringe filtered (0.22 μM) and peptides purified using RP-HPLC as described above. Multiple disulfide isoforms (when present) were separated by a slow gradient of 0.25% solvent B/min. 

#### 3.2.4. Formation of Truncation Analogues by Native Chemical Ligation

NCL was carried out as previously described [18]. Equimolar reduced linear APETx2(Gly1–Ser19)-α-thioester and APETx2(Cys20–Thr39) were dissolved in 3 mL ligation buffer (6 M GnHCl, 0.2 M sodium phosphate, 20 mM TCEP, 30 mM MPAA) to a final concentration of 2 mM, and the pH was adjusted to 7.0. The reaction was carried out under argon and monitored using analytical RP-HPLC (C_18_ column, 2.1 × 250 mm). At the completion of the reaction (16 h), the mixture was loaded onto a preparative RP-HPLC column (Vydac C_18_ column, 10 × 250 mm) and reduced synthetic APETx2_1–39 was eluted with a linear gradient of 5%–28% solvent B over 5 min followed by 28%–58% solvent B over 30 min, at a flow rate of 4 mL/min. Fractions were analysed using RP-HPLC and ESI-MS before being pooled and lyophilised. The same approach was used for the production of APETx2_3–42. Oxidation and purification of the truncation analogues was carried out using the same conditions as described in Section 3.2.3.

#### 3.2.5. Concentration Determination

The concentration of a stock solution of synthetic wild-type APETx2 (sAPETx2_wt) was determined by amino acid analysis (AAA) at the Australian Proteome Analysis Facility (Sydney, NSW, Australia). The area under the RP-HPLC peak (at 214 nm) for all analogues was compared to the area from known amounts of the standard APETx2_wt, which allowed accurate determination of the peptide concentration for all truncation mutants and cyclic analogues.

### 3.3. Nuclear Magnetic Resonance Spectroscopy

Prior to NMR studies, all peptides were lyophilised twice from deionised water. Dry peptide was dissolved in 20 mM phosphate buffer, 5% D_2_O, pH 6.0, then the pH was adjusted to 6.0 with 0.1 M NaOD. NMR samples were filtered in a 0.22 μm centrifuge filter at 14,000 rpm for 10 min. The flow-through was transferred to a 5 mm outer diameter susceptibility matched NMR cell (Shigemi). All data were acquired using a Bruker Avance III 900 MHz spectrometer equipped with a cryoprobe. SOFAST-HMQC spectra were recorded using a repetition time of 0.1 s and 4096 transients per increment (with a total of 30–40 increments). TOCSY spectra were acquired using an MLEV isotropic mixing sequence of 80 ms duration. Spectra were acquired at 298 K and the pH was recorded post NMR acquisition.

### 3.4. Stability Assays

#### 3.4.1. Trypsin Stability Assay

The following protocol was modified from a previous published method [33]: Wild-type APETx2 and cyclic analogues were diluted into 500 μL trypsin buffer (20 mM Tris, 5 mM EDTA, pH 8.0) to a final concentration of 20 μM. Reactions were started by adding 1 μg trypsin to each sample. A 35-μL sample was used for each time point and quenched by mixing with 35 μL 0.2% TFA. Protease digestion was monitored using analytical RP-HPLC. Reactions were incubated at 37 °C and samples were taken at 0, 0.5, 1, 2, 4, 12, 24 and 48 h. All time points were investigated in triplicate.

#### 3.4.2. Simulated Gastric Fluid Assay

The following protocol was modified from a published method [35]: Wild-type APETx2 and cyclic analogues were separately diluted into 350 μL SGF (6.2 mg/mL pepsin, 30 mM NaCl, pH 1.9) to a final peptide concentration of 20 μM. Reactions were started with the addition of the peptides and the mixtures were incubated at 37 °C. 35-μL samples were taken at 0, 15, 30, 45, 60, 120, 240 and 480 min and mixed with 5 μL pepstatin A to quench the reaction (3.3 mg/mL dissolved in ethanol). Progress was monitored using RP-HPLC and each time point was investigated in triplicate.

### 3.5. Electrophysiological Recordings

Oocytes were obtained from *Xenopus laevis* frogs and treated with collagenase (Sigma type I) for defolliculation. cRNA of rat ASIC3 was synthesised using an mMessage mMachine cRNA transcription kit and healthy stage V–VI oocytes injected with 4 ng rat ASIC3 cRNA (40 nL of 100 ng/μL). Oocytes were kept at 17 °C in ND96 solution (96 mM NaCl, 2 mM KCl, 1 mM CaCl_2_, 2 mM MgCl_2_, 5 mM HEPES, 5 mM pyruvic acid, 50 µg/mL gentamicin, 2.5% fetal horse serum, pH 7.4). Membrane currents were recorded 2–6 days post cRNA injection under voltage-clamp (Axoclamp 900 A amplifier, Molecular Devices, Sunnyvale, CA, USA) using two standard glass microelectrodes with resistances of 0.5–1 MΩ when filled with 3 M KCl solution. Data acquisition and analysis were performed using pCLAMP software (Version 10, Molecular Devices, Sunnyvale, CA, USA). Oocytes were clamped at −60 mV with data sampled at 1000 Hz and filtered at 0.01 Hz. All experiments were performed at RT (18–21 °C) in ND96 solution containing 0.1% fatty acid free-bovine serum albumin (BSA). Changes in extracellular pH were induced using a microperfusion system that allowed local and rapid changes of solutions. HEPES was replaced by MES to buffer the pH 6 stimulus solution. Peptide stock solutions were made up in ND96, 0.1% BSA (pH 7.4) to either 1 or 3 μM and serial dilutions were made from these in ND96 solution, 0.1% BSA (pH 7.4). 

## 4. Conclusions

We have shown that backbone cyclisation is a promising strategy for improving the protease resistance of APETx2 and structurally related sea anemone peptides. Our data suggest that the poor protease resistance of APETx2 is conferred predominantly via the peptides’ unprotected *N*- and *C*-termini. However, in the case of APETx2, cyclisation resulted in significant loss of activity against ASIC3. This could be explained by our demonstration that the *N*- and *C*-termini of APETx2 are critical for its inhibition of ASIC3. Further structure-activity relationship studies are needed in order to determine whether APETx2 stability can be improved by cyclisation while maintaining ASIC3 activity. Alternatively, these findings indicate that other more subtle modifications of the termini of APETx2, such as substitution of terminal residues with D-amino acids [36], or use of *N*-terminal pyroglutamate, and/or *C*-terminal amidation [2] could be used to decrease its protease susceptibility.
